# Quality of life among Ethiopian cancer patients

**DOI:** 10.1007/s00520-020-05398-w

**Published:** 2020-03-13

**Authors:** Yemataw Wondie, Andreas Hinz

**Affiliations:** 1 Department of Psychology, University of Gondar, Gondar, Ethiopia; 2 Department of Medical Psychology and Medical Sociology, University of Leipzig, Philipp-Rosenthal-Str. 55, 04103 Leipzig, Germany

**Keywords:** Quality of life, Psychometrics, Reliability, Africa, Illiteracy

## Abstract

**Purpose:**

Cancer is of increasing prevalence in less-developed countries. However, research on the patients’ quality of life (QoL) in these countries is very limited. The aim of this study was to examine QoL of cancer patients in Africa.

**Method:**

A sample of 256 cancer patients treated in an Ethiopian hospital was examined with the European Organization for Research and Treatment of Cancer Quality of Life Questionnaire EORTC QLQ-C30, the Multidimensional Fatigue Inventory, and the Hospital Anxiety and Depression Scale. A group of 1664 German cancer patients served as a comparison group.

**Results:**

Most of the scales of the EORTC QLQ-C30 showed acceptable reliability in the Ethiopian sample. Compared with the German cancer patients, the Ethiopian patients showed lower QoL in most dimensions, especially in financial difficulties, physical functioning, pain, and appetite loss (effect sizes between 0.52 and 0.75). Illiteracy, tumor stage, and treatment (surgery and chemotherapy) were associated with QoL in the Ethiopian sample. QoL was strongly correlated with fatigue, anxiety, and depression.

**Conclusion:**

The EORTC QLQ-C30 is a suitable instrument for measuring QoL in Ethiopia. The detriments in QoL in the Ethiopian patients indicate specific cancer care needs for the patients in a developing country.

## Introduction

Worldwide, the incidence of cancer was estimated to be 18.1 million, and the estimation for cancer mortality was 9.5 million in 2018 [1]. Cancer is the first or second leading cause of death before the age 70 in 91 of 172 countries [2]. In Africa, the incidence and mortality rates are lower than the worldwide average. Though the African proportion of the world population is 16.8%, the shares of cancer incidence and cancer mortality are 5.8% and 7.3%, respectively [2]. With increasing life expectancy, however, the cancer incidence and mortality rates are increasing in Africa [3–6], but it is difficult to obtain reliable epidemiological data in this region.

Quality of life (QoL) has gained increasing relevance in oncological research and treatment in the last decades [7, 8]. One of the most frequently used questionnaires for measuring QoL in cancer patients is the European Organization for Research and Treatment of Cancer Quality of Life Questionnaire EORTC QLQ-C30 [9]. It has been translated into more than 80 languages, and a large number of studies have used this questionnaire in samples of cancer patients, patients suffering from other diseases, and also in the general population. Normative values are available for several countries [10–13]. Most of the studies on QoL in cancer patients have been performed in Western countries.

In less-developed countries, psycho-oncological care and research is limited. Ethiopia ranks 173rd of the 183 countries in the Human Development Index [14]. Several studies have already been performed using the EORTC QLQ-C30 in Ethiopia [15–19]. All these five studies (with one exception) were performed at Tikur Anbessa Referral Hospital in Addis Ababa, the only oncology referral and radiotherapy center of the whole country. These previous studies in Ethiopia did not triangulate the EORTC QLQ-C30 with data of other symptom and psychological distress measures. Therefore, it is relevant to study the QoL of cancer patients treated outside the capital using the EORTC QLQ-C30 and related measures to contribute to the evaluation of the validity of the EORTC QLQ-C30.

To better evaluate the QoL of the Ethiopian patients, we also compared it with that obtained in a large German study to which we had access to the original data. This enabled us to select a sub-sample so the age and gender distributions of both samples were equivalent.

One common problem observed among the Ethiopian studies on cancer patients so far is that many patients are illiterate. In these cases, the study assistants have to read the questions aloud, ask the patients to respond verbally, and mark the response in the questionnaire. It has not been systematically studied whether such a procedure of data collection has a substantial impact on the outcome. Therefore, we analyzed the difference between illiterate and literate patients in responding to the measures we used.

A better understanding of the level of QoL of cancer patients and of factors influencing QoL can lead to raising awareness, promoting the development of policies in cancer care and facilitating better targeted use of limited resources in less-developed countries.

The specific objectives of this study were (a) to determine the level of QoL in Ethiopian cancer patients in comparison with German cancer patients, (b) to test psychometric properties of the questionnaire EORTC QLQ-C30 applied to Ethiopian cancer patients, (c) to analyze the impact of socio-demographic and clinical variables on QoL, and (d) to examine the correlations between the facets of QoL and several other scales.

## Methods

### Ethiopian cancer patients

This study was performed at the University of Gondar Hospital. Gondar is a town in Northwestern Ethiopia with about 300,000 inhabitants. Inclusion criteria for the study were a cancer diagnosis, age 18 and above, and the ability to understand (not to read) the Amharic language. A total of 298 cancer patients were eligible for this study performed between January 2019 and June 2019, of which 256 completed the questionnaire. There were no exclusion criteria concerning tumor entities, disease stage, and illiteracy. Trained research assistants contacted the patients, explained the aims of the study, and asked them to participate and to give informed consent. If the patients were illiterate, the research assistants read the questions aloud, asked the patients to respond verbally, and then marked the response in the questionnaire. The study comprised several questionnaires. Medical data was taken from the medical records of the hospital. The study was conducted in accordance with the Declaration of Helsinki and was approved by the Institutional Review Board of the University of Gondar (Ref. No. O/V/P/RCS/05/1542/2018; dated June 18, 2018).

### German cancer patients

The sample of the German cancer patients was taken from a large psycho-oncological study performed in five study centers in Germany. Detailed information on the sample and the study methods is available in [20]. The ethics committees of all participating study centers approved the study. Data on QoL have already been published [21]. The sample consisted of 1952 males and 2068 females; the mean age was 58.4 years. Due to the differences in the age and gender distribution between the Ethiopian and German sample, we selected a sub-sample of the German cancer patients so the age and gender distributions matched those of the sample from Ethiopia. This procedure resulted in 1664 German cancer patients, 638 males (38.3%), and 1026 females (61.7%). The mean age of this group was 48.0 years, very similar to that of the Ethiopian sample.

Instead of the German sample, we could also have used the mean scores of two broader pooled samples of cancer patients, either 23,553 cancer patients whose mean scores are reported in the EORTC QLQ-C30 reference values manual [22], or 6024 cancer patients included in a pooled analysis [23]. The advantages of these two pooled samples are higher sample sizes with origins from several countries. The disadvantages, however, are that the samples were taken from randomized trials (which means a certain selection bias) and that we had no access to the original data, so we could not select an age- and gender-matched sub-sample. Additionally, the study with the 6024 patients included a high proportion of melanoma patients. For these reasons, we used the German sample as comparison group.

### Instruments

The EORTC QLQ-C30 [9] consists of 30 items assigned to five functioning scales (physical, role, emotional, social, and cognitive functioning), three symptom scales (fatigue, pain, and nausea/vomiting), a two-item global health/QoL scale, and six single items (dyspnea, appetite loss, insomnia, constipation, diarrhea, and financial difficulties). Higher functioning scores indicate better functioning/QoL, whereas higher symptom scores represent more severe symptoms. According to a recommendation of the EORTC Quality of Life Group [24], a sum score of the EORTC QLQ-C30 can be calculated, integrating five functioning scales and eight symptom scales.

Fatigue was measured with the Multidimensional Fatigue Inventory (MFI-20) [25]. This 20-item questionnaire assesses five dimensions of fatigue: general fatigue, physical fatigue, reduced activity, reduced motivation, and mental fatigue. In our study, we used the sum score of all the 20 items. Anxiety and depression were measured with the Hospital Anxiety and Depression Scale (HADS) [26]. This questionnaire consists of 14 items. In this study, we used the HADS total score, integrating across all 14 items [27].

### Statistical analysis

Mean score differences were expressed in terms of Cohen’s effect sizes *d*. Reliability was measured with Cronbach’s *α* coefficient. Two-factor ANOVAs were calculated to test the impact of gender and age group (two categories) on QoL. The impact of further socio-demographic and clinical variables was tested with three-factor ANOVAs with gender and age group as co-variates. Associations between continuous variables were calculated as Pearson correlations. All statistical analyses were performed with SPSS version 24.

## Results

### Socio-demographic characteristics of the sample

In total, 298 patients were eligible for the study. Of these, 256 (86%) were willing to give informed consent and complete the questionnaires. The research assistants checked the questionnaires for missing items which were then completed. The mean age of the sample was 47.9 years (SD = 14.6 years). Further socio-demographic and clinical variables are given in Table 1. A sub-group of 135 patients was illiterate.

**Table 1:** Characteristics of the sample of Ethiopian cancer patients

	Number	Percentage
Gender		
Males	99	38.7
Females	157	61.3
Age category		
18–49 years	127	49.6
≥ 50 years	129	50.4
Marital status		
Single	39	15.2
Married	158	61.7
Divorced	37	14.5
Separate/widowed	22	8.6
Religion		
Christian	230	89.8
Muslim	26	10.2
Education		
Illiterate	135	52.7
Elementary school	38	14.8
Secondary school	31	12.1
Preparatory school	12	4.7
Technical and vocational college	25	9.8
University	15	5.9
Tumor		
Breast	65	25.4
Colon	44	17.2
Non-Hodgkin lymphoma	37	14.5
Cervix uteri	15	5.9
Corpus uteri	9	3.5
Prostate	9	3.5
Colorectal	8	3.1
Thyroid	8	3.1
Lymphocytic lymphoma	6	2.3
Pancreas	6	2.3
Lung	6	2.3
Other	43	16.8
Tumor stage, UICC ^a^		
1	20	7.8
2	66	25.8
3	63	24.6
4	80	31.3
Surgery		
No	125	48.8
Yes	131	51.2
Chemotherapy		
No	109	42.6
Yes	147	57.4
Radiation		
No	234	91.4
Yes	22	8.6

^a^Missing data not reported

### EORTC QLQ-C30 mean scores: comparison between Ethiopian and German cancer patients

The mean scores of the scales are given in Table 2, separately for the Ethiopian and the German sample. The Ethiopian patients reported worse QoL than the German patients; the sum scores yielded an effect size of *d* = − 0.26. Among the scales of the questionnaires, the highest differences in terms of effect sizes were found for financial difficulties (*d* = 0.75) and physical functioning (*d* = − 0.64). The reliability coefficients *α* of the scales were between 0.64 and 0.90 in the Ethiopian sample with the exception of the cognitive functioning scale.

**Table 2:** EORTC QLQ-C30 mean scores and psychometric criteria, comparison between Ethiopia and Germany

	Ethiopia		Germany		Effect size *d*	Ethiopia	Germany
	M	(SD)	M	(SD)		*α*	*α*
EORTC QLQ-C30							
Physical functioning	53.0	(31.9)	70.8	(23.5)	− 0.64	0.85	0.81
Role functioning	51.0	(41.7)	52.0	(34.8)	− 0.03	0.90	0.87
Emotional functioning	64.9	(29.1)	60.8	(26.3)	0.15	0.79	0.85
Cognitive functioning	69.5	(30.0)	71.6	(28.4)	− 0.07	0.46	0.74
Social functioning	49.7	(38.2)	56.0	(33.3)	− 0.18	0.64	0.83
Global health/QoL	54.6	(26.2)	54.4	(22.7)	0.01	0.78	0.89
Fatigue	52.6	(36.1)	50.8	(30.3)	0.05	0.88	0.88
Nausea/vomiting	17.6	(26.3)	12.0	(22.2)	0.23	0.64	0.73
Pain	55.0	(36.2)	36.0	(34.3)	0.54	0.78	0.91
Dyspnea	27.6	(36.4)	27.0	(33.3)	0.02	n.a.	n.a.
Insomnia	38.9	(41.5)	46.5	(37.6)	− 0.19	n.a.	n.a.
Appetite loss	43.4	(40.0)	21.6	(32.1)	0.60	n.a.	n.a.
Constipation	30.9	(37.4)	14.0	(27.9)	0.52	n.a.	n.a.
Diarrhea	8.1	(23.1)	15.8	(28.8)	− 0.30	n.a.	n.a.
Financial difficulties	67.1	(41.2)	37.8	(37.2)	0.75	n.a.	n.a.
Sum score	62.6	(24.2)	68.2	(19.4)	− 0.26	n.a.	n.a.

*d* effect size of the difference between the Ethiopian and the German mean scores, *α* Cronbach’s alpha, *n.a.* not applicable because it is a single-item scale or because the EORTC QLQ-C30 sum score is based on sub-scales and not on items

### Age and gender differences in QoL

**Fig. 1:**
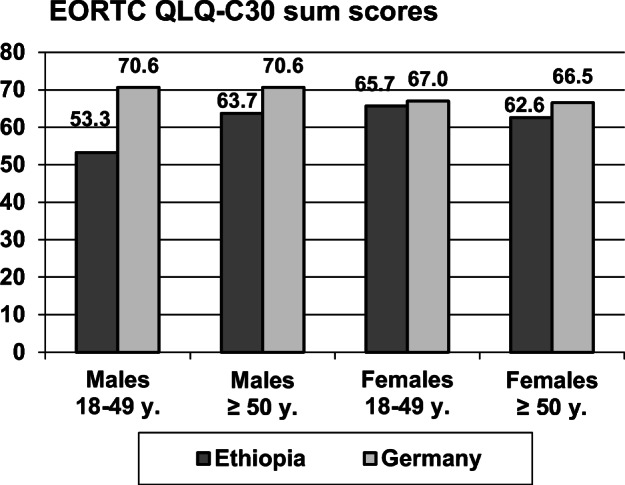
Mean scores of the EORTC QLQ-C30 for the Ethiopian and German sample

Figure 1 illustrates the mean scores of the QoL sum score, broken down by age group and gender. The lowest QoL was found for the Ethiopian young male patients. The ANOVA results of the Ethiopian patients were age group, *F* = 1.32, *p* = 0.251; gender, *F* = 3.14, *p* = 0.077; and age group * gender, *F* = 4.54, *p* = 0.034. The impact of age and gender on the single dimensions of QoL is shown in the following section.

### The impact of socio-demographic and clinical variables on QoL in the Ethiopian sample

Tables 3 and 4 present the impact of socio-demographic and clinical variables on QoL. Table 3 contains the results for the functioning scales, the global health/QoL scale, while Table 4 contains the results for the symptom scales and the sum score.

**Table 3:** EORTC QLQ-C30 functioning scores and global scores in relation to socio-demographic and clinical variables in the Ethiopian sample

		Gender		Age		Education		Tumor type				Tumor stage				Surgery		Chemotherapy	
		Male	Female	≤ 49 years	≥ 50 years	Illiterate	Literate	Breast	Colon	Non-Hodg.	Others	1	2	3	4	No	Yes	No	Yes
*N*		99	157	127	129	135	121	65	44	37	110	20	66	63	80	125	131	109	147
Physical functioning	M	49.3	55.3	54.9	51.1	48.9	57.5	64.0	53.2	49.2	47.6	62.3	61.6	63.1	40.3	43.2	62.3	45.6	58.4
	SD	33.6	30.7	32.3	31.6	33.4	29.6	29.1	32.7	33.0	31.6	28.9	28.7	30.3	31.0	33.4	27.4	34.5	28.8
	Signif.	*p* = 0.121		*p* = 0.934		*p* = 0.173		*p* = 0.061				*p* < 0.001				*p* < 0.001		*p* = 0.007	
Role functioning	M	42.3	56.5	51.6	50.4	44.9	57.7	63.6	51.9	44.1	45.5	55.0	60.3	60.0	40.2	38.5	62.8	39.1	59.7
	SD	41.2	41.2	42.5	41.0	41.1	41.4	40.4	41.8	39.3	42.0	41.2	39.9	40.9	41.8	40.7	39.2	41.6	39.6
	Signif.	*p* = 0.003		*p* = 0.345		*p* = 0.023		*p* = 0.141				*p* = 0.027				*p* < 0.001		*p* < 0.001	
Emotional functioning	M	65.1	64.8	61.5	68.2	60.7	69.6	71.9	67.6	59.4	61.5	68.7	69.4	73.4	59.3	58.7	70.9	60.3	68.3
	SD	29.9	28.6	29.4	28.4	29.2	28.4	28.2	28.3	31.4	28.6	27.5	26.3	28.2	30.5	29.3	27.7	27.8	29.6
	Signif.	*p* = 0.621		*p* = 0.022		*p* = 0.008		*p* = 0.178				*p* = 0.050				*p* < 0.001		*p* = 0.023	
Cognitive functioning	M	67.5	70.7	69.7	69.2	64.9	74.5	73.1	76.5	67.1	65.3	73.3	75.2	75.9	61.7	61.3	77.2	66.8	71.4
	SD	29.8	30.1	30.1	29.9	31.2	27.7	30.4	23.9	30.0	31.4	28.3	28.8	26.6	32.6	30.5	27.4	29.1	30.5
	Signif.	*p* = 0.419		*p* = 0.975		*p* = 0.021		*p* = 0.160				*p* = 0.029				*p* < 0.001		*p* = 0.149	
Social functioning	M	44.3	53.2	49.6	49.9	44.8	55.2	55.4	55.7	40.9	46.9	59.2	61.9	50.0	43.5	41.6	57.5	37.1	59.1
	SD	36.9	38.7	37.6	38.9	36.6	39.3	38.2	34.7	36.8	396	38.4	37.6	37.9	38.9	37.3	37.6	36.8	36.6
	Signif.	*p* = 0.045		*p* = 0.447		*p* = 0.064		*p* = 0.483				*p* = 0.017				*p* = 0.001		*p* < 0.001	
Global health/QoL	M	51.9	56.3	54.8	54.3	50.2	59.4	58.6	57.0	51.8	52.2	62.9	62.5	57.8	47.3	46.2	62.6	45.9	60.9
	SD	27.7	25.1	27.4	24.9	25.6	26.1	25.1	26.8	25.1	26.8	23.9	22.4	26.4	27.3	24.9	24.8	24.3	25.7
	Signif.	*p* = 0.144		*p* = 0.589		*p* = 0.075		*p* = 0.646				*p* = 0.002				*p* < 0.001		*p* < 0.001	

*M* mean, *SD* standard deviation, *Signif* significance level

**Table 4:** EORTC QLQ-C30 symptom scores in relation to socio-demographic and clinical variables in the Ethiopian sample

		Gender		Age		Education		Tumor type				Tumor stage				Surgery	Chemotherapy		
		Male	Female	≤ 49 years	≥ 50 years	Illiterate	Literate	Breast	Colon	Non-Hodg.	Others	1	2	3	4	No	Yes	No	Yes
N		99	157	127	129	135	121	65	44	37	110	20	66	63	80	125	131	109	147
Fatigue	M	57.9	49.2	50.6	54.6	57.4	47.3	40.9	49.0	55.5	60.0	42.2	42.2	42.5	66.7	63.1	42.6	62.6	45.2
	SD	36.3	35.6	36.3	24.9	36.6	34.9	38.1	34.8	37.9	33.1	32.8	34.1	37.0	34.6	34.9	34.4	35.6	34.8
	Signif.	*p* = 0.054		*p* = 0.970		*p* = 0.088		*p* = 0.033				*p* < 0.001				*p* < 0.001		*p* < 0.001	
Nausea/vomiting	M	16.2	18.5	21.4	13.8	17.9	17.2	15.6	18.2	22.5	16.8	15.0	13.6	16.7	19.8	21.3	13.9	20.8	15.2
	SD	24.1	27.6	28.2	23.8	26.8	25.9	25.2	29.2	27.8	25.4	29.1	21.7	28.3	27.3	27.1	25.1	28.9	23.9
	Signif.	*p* = 0.887		*p* = 0.017		*p* = 0.549		*p* = 0.657				*p* = 0.592				*p* = 0.015		*p* = 0.213	
Pain	M	59.4	52.2	53.5	56.4	59.4	50.1	45.4	52.3	58.5	60.6	40.8	44.9	51.0	65.2	68.0	42.6	67.9	45.5
	SD	36.9	35.5	36.7	35.7	35.1	36.8	35.3	36.2	36.6	35.7	32.2	32.1	37.5	37.9	33.8	34.0	33.5	35.2
	Signif.	*p* = 0.079		*p* = 0.715		*p* = 0.146		*p* = 0.097				*p* = 0.004				*p* < 0.001		*p* < 0.001	
Dyspnea	M	29.6	26.3	27.8	27.4	30.6	24.2	25.1	21.2	35.1	29.1	21.7	20.2	24.3	33.3	36.0	19.6	30.6	25.4
	SD	38.9	34.8	37.7	35.2	37.3	35.2	35.9	32.2	40.0	36.9	32.9	30.3	36.5	40.0	38.9	31.9	37.4	35.6
	Signif.	*p* = 0.346		*p* = 0.467		*p* = 0.375		*p* = 0.339				*p* = 0.106				*p* < 0.001		*p* = 0.342	
Insomnia	M	41.4	37.4	42.5	35.4	42.2	35.3	31.8	32.6	39.6	45.5	30.0	27.3	35.4	47.9	45.3	32.8	46.5	33.3
	SD	43.1	40.5	42.1	40.8	42.0	40.9	39.3	41.0	44.3	41.6	37.3	38.7	39.6	43.7	41.9	40.3	42.8	39.8
	Signif.	*p* = 0.276		*p* = 0.106		*p* = 0.179		*p* = 0.106				*p* = 0.015				*p* = 0.006		*p* = 0.003	
Appetite loss	M	44.1	42.9	45.1	41.6	45.4	41.0	35.4	42.4	48.6	46.7	26.7	35.3	39.1	52.5	54.7	32.6	51.1	37.6
	SD	40.3	39.9	41.3	38.9	39.0	41.2	39.0	40.3	43.4	39.1	33.5	37.8	43.4	38.9	39.1	37.9	40.9	38.5
	Signif.	*p* = 0.508		*p* = 0.155		*p* = 0.763		*p* = 0.343				*p* = 0.016				*p* < 0.001		*p* = 0.016	
Constipation	M	31.6	30.4	29.4	32.3	33.8	27.5	29.2	30.3	24.3	34.2	23.3	35.3	25.4	30.0	35.7	26.2	36.4	26.7
	SD	38.2	37.0	36.5	38.4	38.4	36.2	38.9	36.5	34.8	37.9	37.6	40.9	32.6	37.7	38.8	35.6	37.8	36.7
	Signif.	*p* = 0.942		*p* = 0.457		*p* = 0.194		*p* = 0.446				*p* = 0.473				*p* = 0.025		*p* = 0.064	
Diarrhea	M	10.1	6.8	9.7	6.4	8.4	7.7	5.1	14.4	10.8	6.4	6.7	5.5	7.4	12.1	11.5	4.8	5.2	10.2
	SD	25.8	21.3	26.3	19.6	24.7	21.4	21.4	32.5	27.3	17.2	17.4	20.7	22.7	28.2	27.8	17.1	18.2	26.0
	Signif.	*p* = 0.178		*p* = 0.187		*p* = 0.886		*p* = 0.355				*p* = 0.848				*p* = 0.059		*p* = 0.016	
Financial difficulties	M	74.4	62.4	65.3	68.7	70.6	63.1	59.5	55.3	73.9	73.9	60.0	59.1	70.4	67.5	72.3	62.1	77.1	59.6
	SD	37.4	42.8	42.1	40.3	39.7	42.6	43.9	44.3	40.2	37.1	42.7	41.7	41.5	41.7	38.9	42.7	35.9	43.3
	Signif.	*p* = 0.024		*p* = 0.993		*p* = 0.327		*p* = 0.069				*p* = 0.168				*p* = 0.084		*p* = 0.001	
Sum score	M	59.8	64.4	62.1	63.1	59.2	66.5	69.2	65.0	58.9	59.0	70.2	69.5	67.7	55.3	54.4	70.4	56.0	67.5
	SD	24.8	23.8	24.3	24.2	24.3	23.6	25.3	21.8	25.9	23.3	21.9	21.1	24.5	25.2	23.8	22.0	24.0	23.2
	Signif.	*p* = 0.077		*p* = 0.251		*p* = 0.044		*p* = 0.105				*p* = 0.002				*p* < 0.001		*p* < 0.001	

*M* mean, *SD* standard deviation, *Signif.* significance level

Males reported lower levels of role and social functioning and more financial difficulties than females. Concerning age, there was only one significant difference: older patients reported better emotional functioning than younger patients. Illiterate patients showed worse QoL than literate patients in all of the 15 dimensions; the differences were statistically significant in three of the dimensions. Concerning tumor type, there was only one significant difference in the fatigue level. Tumor stage was strongly associated with multiple dimensions of QoL. While the mean scores for stages 1 to 3 did not show large differences, patients with stage 4 reported lowest functioning scores and highest levels of symptoms in most of the scales. Patients receiving surgery or chemotherapy were characterized by better QoL than those patients not receiving those treatments.

### Correlations between QoL scales and other scales

Correlations within the Ethiopian sample between the scales of the EORTC QLQ-C30 and the two-item global health/QoL scale, the MFI-20 sum score, and the sum score of the HADS are presented in Table 5. The global assessment of health/QoL was most strongly associated with pain (*r* = − 0.59) and fatigue (*r* = − 0.58). The sum score of the fatigue questionnaire MFI-20 correlated most highly with the fatigue scale of the EORTC QLQ-C30 (*r* = 0.74), and anxiety and depression (HADS total scale) were most strongly associated with the EORTC QLQ-C30 scales emotional functioning, fatigue, and pain (*r* = 0.65 each).

**Table 5:** Correlations between the EORTC QLQ-C30 scales and the Global health/QoL scale, the MFI-20 sum score, and the HADS sum score

	QLQ-C30 Global health/QoL	MFI-20 sum score	HADS sum score
Physical functioning	.51 ***	− .67 ***	− .57 ***
Role functioning	.54 ***	− .66 ***	− .59 ***
Emotional functioning	.51 ***	− .56 ***	− .65 ***
Cognitive functioning	.48 ***	− .61 ***	− .64 ***
Social functioning	.52 ***	− .57 ***	− .59 ***
Global health/QoL		− .56 ***	− .55 ***
Fatigue	− .58 ***	.74 ***	.65 ***
Nausea/vomiting	− .27 ***	.33 ***	.39 ***
Pain	− .59 ***	.70 ***	.65 ***
Dyspnea	− .36 ***	.39 ***	.40 ***
Insomnia	− .48 ***	.45 ***	.49 ***
Appetite loss	− .42 ***	.47 ***	.47 ***
Constipation	− .30 ***	.27 ***	.34 ***
Diarrhea	− .18 **	.12 ns	.15 *
Financial difficulties	− .41 ***	.39 ***	.43 ***
Sum score	.65 ***	− .73 ***	− .73 ***

*ns* not significant

*p < 0.05; **p < 0.01;***p < 0.001

## Discussion

Most of the EORTC QLQ-C30 scales proved to have sufficient reliability. Due to the small number of items per scale, it is not surprising that the EORTC QLQ-C30 scales did not reach the levels of reliability achieved by other questionnaires with more items per scale. The reliability of the cognitive functioning scale (*α* = 0.46) was very low; however, another Ethiopian study [16] and a study from Tanzania [28] reported even lower coefficients, both below 0.40. The relatively low alpha coefficients for cognitive functioning, social functioning, and nausea/vomiting indicate there is some insecurity in the measurement on the individual level; however, on a group level, assessments are justified.

The comparison between the Ethiopian and the German cancer patients shows the burden is higher in the Ethiopian sample in most of the scales. All functioning scales (except emotional functioning) showed lower mean scores in the Ethiopian sample and all symptom scales (except insomnia and diarrhea) showed higher scores for the patients in Ethiopia. The most pronounced differences were found for financial difficulties (*d* = 0.74). Two other studies with Ethiopian cancer patients [17, 29] also reported high mean scores in these scales (64.8 and 69.9, respectively), similar to the mean of our Ethiopian study (67.1) and markedly higher than the mean score obtained in Germany (37.8). This difference illustrates that due to the lack of a health insurance system, the financial burden of cancer patients is considerable and this non-clinical factor is relevant for the QoL of patients in developing countries. In the Tanzanian study, the mean score of financial difficulties (84.3) was even higher than that in Ethiopia. Appetite loss and pain were also more pronounced in the Ethiopian sample when compared with the German one with effect sizes of *d* = 0.60 and *d* = 0.54, respectively. This is possibly due to the lower availability of adequate medication and lack of pain management interventions in Ethiopia.

However, the meaningfulness of the comparisons between German and Ethiopian cancer patients is limited by the fact that no Ethiopian normative values exist. Thus, we cannot conclude which part of the differences is caused by different medical care conditions and which part is caused by different response behavior in the general population in these countries. Normative studies from developing countries would help clarify this problem.

If we had considered the data of the EORTC QLQ-C30 reference values manual [22] instead of the German data, the differences to the Ethiopian mean scores would have been even larger. With the exception of the constipation scale, the manual reported better QoL than that of the German cancer patients’ sample, making the difference to the Ethiopian sample even greater. For the physical functioning scale (M = 76.7 in the manual), e.g., the effect size increases from *d* = − 0.64 (German vs. Ethiopian sample) to *d* = − 0.86 (manual vs. Ethiopian sample). However, the participants of the samples considered in the manual are of other age and gender distribution than the Ethiopian sample, and being included in a clinical trial (patients of the manual) might also produce a certain selection bias since patients with severe diseases are less likely to be included in clinical trials.

It is interesting to note that despite the differences between the Ethiopian and the German sample in the sum score and in several of the functioning and symptom scales, the assessments of the two-item global health/quality of life scale were nearly equal in both samples, M = 54.6 in Ethiopia and M = 54.4 in Germany. A study with comparisons between cancer patients and the general population showed the patients reported severe detriments in several dimensions of QoL when compared with the general population, but they nevertheless rated their global QoL as relatively good [30]. A global assessment of one’s health/QoL is therefore not identical with the sum of the assessments of specific symptoms and functioning facets. One implication of this fact is that examinations of QoL in cancer patients should not be restricted to such a global assessment of health or QoL since the differences found in this global scale can underestimate the real differences.

Illiterate patients reported lower QoL mean sum scores than those who could read and write. However, this group difference was not statistically significant. The differences between the cancer types also failed to reach the significance level. However, we observed a clear and nearly linear association between tumor stage and QoL (*p* < 0.001). Patients with tumor stage 4 reported the most severe detriments, especially in the domains physical functioning and fatigue. While this relationship is easily understandable and in line with other studies [29, 31], it is interesting to note that patients receiving surgery or chemotherapy reported better QoL than those patients not receiving that treatment. This relationship cannot be attributed to group differences in the distribution of the tumor stages: the proportion of patients receiving chemotherapy varied between 58 and 62% in the four stage groups, and concerning surgery there was a (non-linear) association between receiving surgery and tumor stage with the highest surgery proportions for stage 2 (64%) and stage 3 (60%), while the proportions were lower for stage 1 (55%) and stage 4 (39%). The result of better QoL in patients receiving certain treatment was not found in previous studies from the middle-income countries Malaysia [32] and Taiwan [33]. These differences could be explained partly by the low level of economic standards of the Ethiopian cancer patients who might not be able to get the necessary treatment when needed, which could have contributed to their higher level of symptoms. On the other hand, receiving surgery or chemotherapy might have given the Ethiopian cancer patients the feeling of getting relief from such symptoms. Further research is needed to explain this effect.

The correlations between the specific EORTC QLQ-C30 scales and global health/QoL scale (Table 5) show that all specific aspects of QoL contributed to the global assessment of the patients’ health/QoL, whereby pain (*r* = − 0.59) and fatigue (*r* = 0.58) had the most relevant impact. A cross-cultural study [34] analyzed the relationship between the specific QoL scales and the global health/QoL scale in 10 different regions of the world. In seven of these 10 regions, fatigue correlated most highly with global health/QoL, while emotional functioning and pain were the next relevant dimensions. Unfortunately, Africa was not included in this analysis.

The high correlation between the MFI-20 fatigue total score and the EORTC QLQ-C30 fatigue scale (*r* = 0.74) indicates convergent validity of the fatigue scale. The correlation between the HADS total score (anxiety and depression) and the EORTC QLQ-C30 scales was highest for the scales emotional functioning (*r* = − 0.65), fatigue (*r* = 0.65), pain (*r* = 0.65), and cognitive functioning (*r* = − 0.64). Together with the high correlation between fatigue and global health/QoL, the results show that fatigue (an overwhelming feeling of exhaustion and tiredness) is a severe problem which strongly impairs mental well-being and QoL. The crucial role of cancer-related fatigue has been documented in multiple studies [35]. Physicians who sometimes tend to overlook this symptom should be aware of its relevance.

This study has several limitations. It was performed in one hospital in Gondar, Ethiopia, and hence, the generalizability to other clinics or countries remains open. About half the patients could not read the questionnaire. The impact of reading the questions aloud by the study assistants on the response behavior has not been systematically analyzed. Though other studies performed in developing countries also used this method, there is no systematic comparison of the psychometric properties of the questionnaires between illiterate and literate patients. Further research should clarify whether there are such systematic differences, irrespective of the specific content of the questionnaires. Several clinical factors were not independent of each other; therefore, the impact of these factors on the QoL assessments may partly be mediated by other factors, even if the significance tests of Tables 3 and 4 considered the potential confounders age and gender. The German sample of cancer patients was designed to be roughly representative of all cancer patients in Germany; however, the data cannot be generalized to “Western countries.” Even within Europe, there are differences in the EORTC QLQ-C30 mean scores of the general population, the means of the global health/QoL scale range from 60.0 in Poland to 77.4 in the Netherlands [10].

In summary, the EORTC QLQ-C30 proved to be fairly applicable to Ethiopian cancer patients speaking the Amharic language, even when patients were illiterate. The differences between the Ethiopian and the German mean scores (financial difficulties, physical functioning, pain, fatigue, appetite loss) show specific cancer care needs for the population in a developing country.
